# Facilitating Advance Care Planning for Patients With Severe COPD: A Qualitative Study of Experiences and Perceptions of Community Physicians, Nurses, and Allied Health Professionals

**DOI:** 10.1097/NHH.0000000000000945

**Published:** 2021-03-04

**Authors:** Yoshihisa Hirakawa, Kaoruko Aita, Mitsunori Nishikawa, Hidenori Arai, Hisayuki Miura

**Affiliations:** **Yoshihisa Hirakawa, MD, PhD**, is an Associate Professor, Graduate School of Medicine, Nagoya University, Nagoya, Aichi, Japan.; **Kaoruko Aita, PhD**, is a Project Professor, Graduate School of Humanities and Sociology, University of Tokyo, Tokyo, Japan.; **Mitsunori Nishikawa, MD**, is a Member, Department of Palliative Care, National Center for Geriatrics and Gerontology, Obu, Aichi, Japan.; **Hidenori Arai, MD, PhD**, is President, National Center for Geriatrics and Gerontology, Obu, Aichi, Japan.; **Hisayuki Miura, MD, PhD,** is Head, Department of Home Care and Regional Liaison Promotion, National Center for Geriatrics and Gerontology, Obu, Aichi, Japan.

## Abstract

Chronic obstructive pulmonary disease (COPD) is a major cause of morbidity worldwide. Patients with severe COPD often fail to receive adequate palliative care and are subject to undesired hospital transfers and cardiopulmonary resuscitation. Although promoting advance care planning (ACP) in the community can help ensure the optimal delivery of palliative care for patients with COPD, the key challenges to routinely implementing ACP are not known. The aim of this study was to identify the perception of healthcare professionals with regard to ACP for adults living with severe COPD and the challenges to facilitating ACP. A multicenter qualitative study design was used. In-depth semistructured interviews were held involving 38 healthcare professionals from 19 institutions in Japan. Text data were analyzed by content analysis. Five main themes capturing the challenges to routine implementation of ACP were identified: daily decision-making; sense of ethical decision-making; in-depth interviewing skills; collaborative information sharing among team members; and knowledge dissemination regarding ACP. The model demonstrates the complexity inherent in ACP facilitation for community-dwelling adults with severe COPD, with all the elements required for successful ACP implementation. We recommend an approach that recognizes the importance of stakeholder education, particularly educating professionals to develop the knowledge, attitudes, and skills required for ACP facilitation: in-depth interviewing, collaborative information sharing, and ethical analysis, focusing on decision-making concerning everyday life support.

**Figure:**
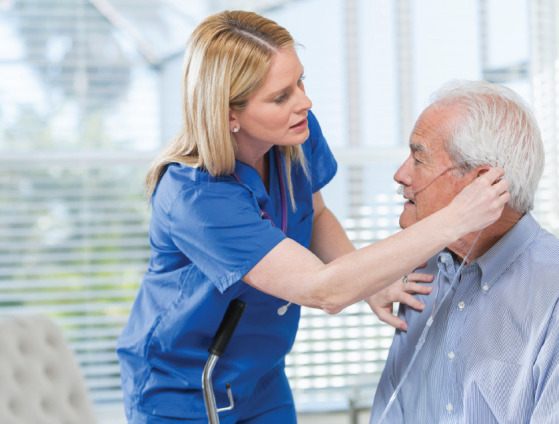
No caption available.

Chronic obstructive pulmonary disease (COPD) is a major cause of morbidity, and is expected to become the third leading cause of death worldwide by 2030 (WHO, 2008). It is a progressive and debilitating illness characterized by acute exacerbations of symptoms and an increased difficulty in performing activities of daily living (Barusso et al., 2015; Gardiner et al., 2010). This places patients with severe COPD at an increased risk of burden and sudden death (Gardiner et al.).

In the community, palliative care for severe COPD is provided by a long-term care team composed of primary care providers, nurses, case managers, social workers, and other home care providers. These care team members, however, are not palliative care specialists (Gardiner et al., 2010). The health of patients with COPD often deteriorates abruptly and unpredictably as they approach death; as a result, there are few opportunities for long-term care teams to hold end-of-life care discussions to clarify prognosis, explain illness trajectory, and adopt advance care planning (ACP) options that respect the values, preferences, and goals of patients and families (Patel et al., 2012). Therefore, patients with severe COPD often fail to receive adequate palliative care and are subject to undesired hospital transfers and cardiopulmonary resuscitation.

Although ACP has become a mainstay in cancer care, it is still challenging for long-term care teams to routinely implement ACP for noncancer patients (Glaudemans et al., 2015), especially for older people with COPD who experience severe morbidity for extended periods of time, and suffer acutely from impaired activities of daily living and compromised physical, social, and spiritual functioning (Andreas & Alt-Epping, 2018).

Previous studies have underscored the existence of a number of barriers to the routine implementation of ACP in COPD and other noncancer chronic diseases (Patel et al., 2012). Some researchers suggested that long-term care teams were unaware of the life-limiting nature of the disease and that death was imminent (Gardiner et al., 2010; Patel et al.). Others suggested that noncancer patients were unlikely to openly express a wish for help because they did not want to bother their family and physician (Fukui et al., 2013; Hirakawa et al., 2017). In other cases, physicians were hesitant to discuss important issues due to lack of time and preparation until it was too late to react (Gardiner et al.; Vermylen et al., 2015). Thus, there are missed opportunities for ACP for COPD patients.

Routinely implementing ACP for adults living with severe COPD can help ensure the optimal delivery of palliative care. However, it is still unclear what challenges to implementing ACP for such patients exist, or what types of roles long-term care teams should play to address these challenges. The aim of this study was to identify the perception of healthcare professionals with regard to ACP for adults living with severe COPD and the challenges to facilitating ACP.

## Method

### Study Design and Target

Given the limited information available on ACP for severe COPD in Japan, we opted for a qualitative approach for this study. A qualitative study is used to carry out an in-depth investigation into a specific situation and its impact on a particular group. We conducted in-depth semistructured interviews with healthcare professionals, including nurses, care managers, and social workers caring for adults with severe COPD from 19 institutions in Japan.

We first conducted key informant interviews in an eight-person focus group discussion. The recruitment of key informants which targeted nurses, care managers, and social workers was done via convenience sampling, while considering their knowledge and experience concerning ACP practice. The characteristics of the key informants are listed in Table 1. Subsequently, we purposively recruited 30 participants from urban, suburban, and rural areas of Akita, Tokyo, Nagoya, and Tokushima, and conducted individual interviews. The participants were selected from among the authors' acquaintances, all of whom had extensive experience of palliative care service provision for people with nonmalignant respiratory disease and their family caregivers. The characteristics of the participants are shown in Table 2.

**Table 1.** Characteristics of Key Informants

Code	Professional	Specialty	Gender	Age Range	Other Relevant Information
K1	Physician	Geriatric medicine	Male	35-40	A lot of clinical experience in both hospital and home care
K2	Nurse	Home care	Male	50-52	None
K3	Nurse	Home care	Female	45-50	None
K4	Nurse	Home care	Female	45-50	None
K5	Nurse	Home care	Female	40-45	None
K6	Care manager	None	Male	45-50	A lot of experience in community social work practice
K7	Care manager	None	Female	50-55	None
K8	Care manager	None	Female	40-45	A lot of experience in long-term care insurance consultations

**Table 2.** Characteristics of Participants

Code	Professional	City	Gender	Age Range	Other Relevant Information
P1	Physician	Urban Akita	Male	55-60	Palliative care physician with a lot of experience in nursing education
P2	Physician	Suburban Tokyo	Male	55-60	Clinician with a lot of experience in respiratory medicine and dementia care
P3	Physician	Suburban Tokyo	Female	45-50	Clinician undergoing clinical training for home palliative care
P4	Physician	Urban Nagoya	Male	45-50	Geriatrician with a lot of experience in advance care planning
P5	Physician	Rural Nagoya	Male	50-55	Clinician with a lot of experience in home palliative care
P6	Physician	Suburban Tokushima	Male	40-45	Hospitalist working in general internal medicine
P7	Nurse	Urban Akita	Female	50-55	Chief nurse working at outpatient department
P8	Nurse	Suburban Tokyo	Female	45-50	Home-visit nurse with a lot of experience in practice of advance care planning
P9	Nurse	Suburban Tokyo	Female	45-50	Home-visit nurse with a lot of experience in practice of advance care planning
P10	Nurse	Suburban Tokyo	Female	45-50	Home-visit nurse with a lot of experience in practice of advance care planning
P11	Nurse	Suburban Tokyo	Female	30-35	Home-visit nurse with a lot of experience in practice of advance care planning
P12	Nurse	Urban Nagoya	Female	45-50	Teacher in nurse education
P13	Nurse	Urban Nagoya	Female	45-50	Home-visit nurse with a lot of experience in practice of advance care planning
P14	Nurse	Urban Nagoya	Female	45-50	Home-visit nurse with a lot of experience in practice of advance care planning
P15	Nurse	Urban Nagoya	Female	40-45	Teacher in nurse education
P16	Nurse	Rural Nagoya	Male	50-55	Home-visit nurse with a lot of experience in practice of advance care planning
P17	Nurse	Rural Nagoya	Female	65-70	Home-visit nurse with a lot of experience in practice of advance care planning
P18	Nurse	Rural Nagoya	Female	60-65	Home-visit nurse with a lot of experience in social work
P19	Nurse	Rural Nagoya	Female	60-65	Home-visit nurse with a lot of experience in practice of advance care planning
P20	Nurse	Rural Nagoya	Female	50-55	Home-visit nurse with a lot of experience in practice of advance care planning
P21	Nurse	Rural Nagoya	Female	50-55	Home-visit nurse with a lot of experience in practice of advance care planning
P22	Nurse	Rural Nagoya	Female	45-50	Home-visit nurse with a lot of experience in practice of advance care planning
P23	Nurse	Rural Nagoya	Female	45-50	Home-visit nurse with a lot of experience in practice of advance care planning
P24	Nurse	Rural Nagoya	Female	40-45	Home-visit nurse with a lot of experience in practice of advance care planning
P25	Nurse	Rural Tokushima	Female	35-40	Home-visit nurse with a lot of experience in respiratory care
P26	Social worker	Suburban Nagoya	Female	50-55	Medical social worker working at hospital
P27	Social worker	Rural Nagoya	Female	50-55	Medical social worker working at hospital
P28	Social worker	Rural Nagoya	Female	40-45	Medical social worker working at hospital
P29	Social worker	Suburban Tokushima	Male	35-40	Medical social worker working at the same hospital as P6
P30	Care manager	Rural Tokushima	Female	40-45	Director of general home care center

### Data Collection

A semistructured interview guide was developed based on the PRECEDE-PROCEED model, which is a comprehensive structure for assessing health needs for designing, implementing, and evaluating health promotion (Ghaffarifar et al., 2015). The main topics were: (a) behavior and lifestyle related to ACP in COPD; (b) environmental assessment of ACP in COPD; and (c) predisposing, reinforcing, or enabling factors of ACP in COPD.

Key informant interviews and in-depth interviews were conducted between October and November 2019. Key informant interviews were conducted in a conference room and took approximately 60 to 90 minutes. The informants were first asked about the topics in the guide; this was followed by inductive probing based on the generated ideas. In-depth interviews were conducted at the participants' place of work or in a rental conference room, and took 90 to 120 minutes. The key informant interviews and in-depth interviews were all facilitated by the first author, a geriatrician with extensive experience in qualitative research.

### Data Analysis

The interviews were audio-recorded and transcribed verbatim. The first author read the transcriptions repeatedly to get well acquainted with the data. We then conducted line-by-line coding, where pieces of data were segmented and condensed into individual sentences (meaning units). Then, the emergent meaning units were discussed among the authors until consensus was reached. The grouping process involved reading and comparing individual meaning units in order to cluster similar units into categories and inductively formulate themes.

The study was approved by the Bioethics Review Committee of Nagoya University School of Medicine, Japan (approval No. 2015-0444). All participants were informed of the objectives of the study, and they were also notified of their rights to withdraw from the study at any time and to skip questions or topics they did not wish to discuss. Written informed consents were obtained and documented from all participants.

## Results

Five main themes capturing the challenges of routine implementation of ACP were ultimately identified: a) daily decision-making; b) sense of ethical decision-making; c) in-depth interviewing skills; d) collaborative information sharing among team members; and e) knowledge dissemination regarding ACP. They are presented below with illustrating quotes.

### Daily Decision-Making

Although ACP discussions should normally focus on eliciting patients' values, wishes and beliefs, our informants' responses revealed that current ACP practices are mainly centered on decision-making about future medical care. However, some nonphysician participants highlighted that decision-making also concerned the patients' capacity to perform everyday activities. This result implied that educational interventions to cultivate physicians' and other healthcare professionals' insight into COPD patients' daily life functioning were needed. For example:

“Many clients say that if they ever became unable to go to the restroom or to take a bath due to respiratory failure, they would prefer to die.” (P20, nurse)

“Home-visit nurses focus on supporting their clients' wishes regarding activities of daily living, like eating, bathing, and toileting, rather than on life-sustaining treatments.” (P13, nurse)

### Sense of Ethical Decision-Making

During palliative care of severe COPD, a number of ethical dilemmas may arise due to communication breakdowns, actions compromising patient autonomy, ineffective dyspnea management, nonbeneficial care, and undesired hospital transfers. Experienced clinicians can navigate these ethical dilemmas by offering the best possible care while also allowing patients the opportunity to make choices about how to live with dignity and what type of care they wish to receive. However, the participants indicated that because care managers were less experienced in tackling ethical issues related to end of life, they were more likely to simply act as spokespersons for their patients and their families. Ethical skills, including ethical reasoning and reflection, value-aware communication skills, and informed decision-making skills should be taught to care managers.

“Some care managers are reluctant to transfer patients to the hospital if they wish to die at home. We would prefer that care managers contact us when medical care is required.” (P9, nurse)

“From an ethical standpoint, good care managers consider both the end-of-life care options that the clients and families choose, and the motivations behind these decisions.” (P1, physician)

When patients lack proper decision-making capacity, family members may try to control aspects of their loved ones' lives, including end-of-life decisions. Physicians and nurses routinely consider family members as “key referents” and conduct end-of-life care discussions with them before giving patients informed choices. Relying too heavily on families for decision-making might hamper patients' autonomy and place undue stress on families.

“Physicians and nurses often leave decision-making regarding hospitalization and life-sustaining treatments to patients' relatives even when family members are estranged.” (P26, social worker)

“Some families ask us to continue to feed their loved ones even when they experience dysphasia and are at risk of choking or developing pneumonia.” (P4, physician)

### In-Depth Interviewing Skills

Successful ACP requires in-depth discussions with individuals capable of reflecting on themselves. If COPD patients are unaware of their precarious condition, ACP discussions are likely to be futile. On the other hand, COPD patients with severe dyspnea may feel like they're about to die and associate ACP discussions with a death sentence. Thus, conducting a successful ACP discussion with COPD patients poses an important challenge for physicians, nurses, and care managers.

“Physician-led ACP, which is commonly practiced, bears resemblance with informed consent decisions.” (P17, nurse)

“To ensure quality person-centered care, it is important to confirm the patients' care preferences and understand the reasons behind these choices. I think that health care professionals engaging in ACP communication must develop in-depth interview skills.” (P7, nurse)

“Patients experiencing severe dyspnea may feel like they're about to die, so when ACP discussions are initiated with them at that time, they might think that they are being handed a death sentence; this is why I prefer to wait to talk about ACP until symptoms disappear or improve.” (P19, nurse)

### Collaborative Information Sharing Among Team Members

Hospital physicians often assume that they cannot risk sending severe COPD patients home if they do not have enough time to discuss ACP with them and their family. Participants underscored that close and continuous hospital–primary care collaboration related to ACP enabled patients with severe COPD to spend their last moments of life at home as they hoped. Additionally, lack of hospital–primary care collaboration is likely to prevent primary palliative care teams from taking over ACP and implementing symptom management plans made during hospitalization. Study participants were aware of the importance of the mediation role of palliative care specialists in this collaborative endeavor.

“Upon hospital admission, I usually inform doctors that my clients have formulated advance directives so that they are aware of the end-of-life wishes of my clients and their family.” (K7, care manager)

“ACP handover is essential to ensure continuity of care throughout the hospital-to-home or home-to-hospital transition for patients with COPD. In addition, ACP documents should originally be made in a home care setting because physicians and nurses often find it difficult to adequately document ACP in intensive care.” (P22, nurse)

The lack of time to discuss key issues with patients is a fundamental barrier to adequate ACP. Two participants (P10, nurse; P23, nurse) suggested that individual team members collect qualitative data on their patients' end-of-life wishes to be shared collectively: each team member could take daily notes of the information gathered through small talk with individual patients and share these with other team members, and deductions could thus jointly be made regarding the patients' end-of-life wishes.

The greatest challenge to shared decision-making for end-of-life care in COPD is the unpredictable disease trajectory. Participants expressed concerns about the inconsistency in individual team members' understanding of ACP results. Documented life-sustaining treatment, resuscitation and intubation, or home death preferences in ACP are not specific enough for physicians and nurses to provide appropriate home palliative care in line with the patients' wishes and values.

“If a person with severe COPD wishes to die at home, health care professionals believe that avoiding life-sustaining treatment and providing end-of-life care for pain and other symptoms are appropriate measures to take for this patient. However, I know there are primary care physicians who continue to provide aggressive or hospital-level care.” (P9, nurse)

“Certain clients with severe COPD who had originally wished to die at home, later chose to be hospitalized when they began experiencing unbearably severe dyspnea.” (P21, nurse)

“When long-term feeding is medically required, withholding or withdrawing tube feeding may be considered as undertreatment and an ethical issue. However, in some cases, family members view tube feeding as life support, and as against their loved ones' wishes.” (K1, physician)

### Knowledge Dissemination Regarding ACP

Given that ACP content covers medical treatment as well as personal values and preferences, it would be best for everyone in the community, including healthcare professionals, and older people and their families, to be well informed about ACP. However, study participants underscored the magnitude of challenges to improve knowledge and understanding of ACP among physicians, nurses, and other allied health professionals as well as lay persons in the community.

“Knowledge about and attitude toward ACP vary widely among physicians.” (P27, social worker)

“Many care managers view ACP as small talk about irrelevant topics such as favorite foods.” (P30, care manager)

## Discussion

**Figure 1.**
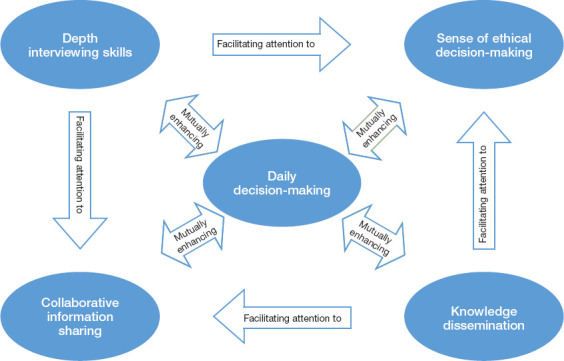
Conceptual model of interlinking elements to facilitate ACP for community-dwelling COPD patients

The main behavioral components, factors, and requirements necessary to routinely implement ACP in severe COPD were identified in this study and are represented conceptually in Figure 1. The model demonstrates the complexity inherent in ACP facilitation for community-dwelling adults with severe COPD, with all the elements required for successful ACP implementation. It recommends an approach that recognizes the importance of stakeholder education, particularly educating professionals to develop the knowledge, attitudes, and skills required for ACP facilitation: in-depth interviewing, collaborative information sharing, and ethical analysis, focusing on decision-making concerning everyday support. Greater knowledge of ACP would facilitate long-term care team discussions with COPD patients and families. Recognition of COPD patient preferences can be enhanced by delving more deeply into their preferences for everyday living. Educational opportunities focusing on ACP are encouraged for physicians, nurses, and other clinicians.

The results suggested that decision-making concerning daily living activities should be taken into account in ACP discussions. Previous studies have revealed that patients with COPD have a high symptom burden with a heavy impact on quality of life and social functioning (Gardiner et al., 2010; Patel et al., 2012). When compared with patients with advanced lung cancer, patients with COPD were found to have significantly more difficulty performing activities of daily living (ADLs; Gardiner et al.). In general, increased ADLs can exacerbate breathing difficulty in people with COPD and even increase the risk of a sudden exacerbation (Patel et al.). However, information provision regarding COPD trajectory is often lacking, and the prognosis implications or the impact of COPD on ADLs are not routinely discussed (Meehan et al., 2019; Patel et al.; Smallwood et al., 2018). Moreover, although ACP generally includes deciding what life-sustaining treatment the patients would prefer and sharing the patients' personal values and beliefs with their families, ACP forms normally do not address patient wishes in terms of ADLs under severe conditions such as exacerbated dyspnea in COPD (Bridges et al., 2018; Gazarian et al., 2019; Hirakawa et al., 2017; Rietjens et al., 2017; Sudore et al., 2017).

Although it is widely accepted that a collaborative approach between hospitals and community stakeholder organizations is key to building a primary palliative care team (Buurman et al., 2010; McCurdy, 2012; Meier, 2011), our results suggested that developing and sustaining seamless, multidisciplinary, and multifacility collaborations in ACP remains challenging for many long-term care teams. First, this study underscored that sharing ACP for patients with COPD across care settings is essential to provide quality primary palliative care in the community. However, a number of previous studies suggested that sharing relevant information on admission to, and discharge from, different care settings was generally poorly conducted (Baldwin et al., 2018; Labson, 2015; McCurdy). Second, this study recommended the implementation of a team-based information gathering process that involves the collection of pieces of information about patients' wishes and preferences at end of life. This approach would provide a coherent foundation on which to base ACP meetings between team members and patients and families, even when lack of time is an issue (Furman et al., 2018; Pillay et al., 2016; Weppner et al., 2016). Third, this study suggested that sharing detailed ACP as a team can reduce the likelihood of disagreements, ethical dilemma, and conflict between family members and within healthcare teams, particularly when the patient's wishes for treatment or hospitalization are unknown or unclear. Previous studies suggested that recording concrete, specific, and relevant information about the patients' treatment preferences is important in determining whether treatments are consistent with their wishes (Cox et al., 2011; Unroe et al., 2016; Walker et al., 2011).

Over the last decade, the focus of ACP has shifted from a document-driven, decision-oriented form to a developmental, person-centered discussion process (David et al., 2019). ACP is seen as an important strategy to improve end-of-life communication and the quality of life of patients and their families (Zwakman, 2018). Therefore, in ACP discussions, in-depth interviews are used to focus on a detailed account of the life of a patient (Briggs, 2004). Although a number of comprehensive ACP training courses have been developed to expand the workforce of skilled professionals trained to promote and facilitate ACP in the community, most of these focused on initiating ACP discussions but not on in-depth interviewing skills (Chan et al., 2019).

Although numerous studies have dealt with ACP implementation around the globe (Bristowe et al., 2014; Chan et al., 2019; Gleeson et al., 2019; McGlade et al., 2017; Sudore et al., 2015), few studies have investigated Japanese settings. This study emphasized the dissemination of knowledge and skills among the various stakeholders in the community: lay people, patients with COPD, families, physicians, nurses, and allied health professionals as facilitators to the implementation of ACP in Japanese community care for COPD. Even though the Japanese government has made serious efforts to facilitate the normalization of ACP discussions and promoted the dissemination of information and knowledge (Tanaka et al., 2015), ACP is still poorly recognized by healthcare professionals as well as lay persons (Hirakawa et al., 2018; Inoue et al., 2019; Tanaka et al.).

Our study focused on a small number of participants due to a lack of data on physicians, nurses, and allied health professionals who are knowledgeable about both COPD and ACP. The qualitative data were not collected from nonexpert healthcare professionals, and was thus possibly limited in terms of diversity and variability of responses. Although the first author has extensive experience with qualitative research interviews, there may have been a social desirability bias at play because participants knew the research teams were composed of respiratory specialists and/or ACP research experts, and because most of the interviews took place at the participants' workplace. To enhance diversity and inclusion, qualitative data were collected from both rural and urban areas. However, convenience sampling is the least rigorous approach to defining a study sample and may result in low accuracy, poor representativeness, low credibility, and lack of transferability of study results (Johnson et al., 2020). Although patients with COPD normally experience a wide range of physical and psychological symptoms, the specific symptoms related to COPD often go unrecognized by healthcare professionals. Also, the illness trajectory of COPD patients is more likely to vary widely as compared with patients with pneumonia, asthma, and lung cancer, which is more predictable. The characteristic variety might have led to the participants' lack of accurate and comprehensive understanding of COPD. Finally, although the authors focused on ACP for patients with COPD, the participants were unable to identify the facilitators and barriers peculiar to COPD to the implementation of ACP. Therefore, the qualitative data collected in this study might have included general information about ACP in addition to information specific to COPD.

## Conclusion

This study identified the main behavioral components, factors, and requirements necessary to facilitate ACP for community-dwelling COPD patients, and proposed a conceptual model emphasizing stakeholder education, particularly targeting professionals, to develop the knowledge, attitude, and skills required for ACP facilitation: in-depth interview skills, collaborative information sharing, and ethical analysis, focusing on decision-making about everyday life support.
